# Supplementary material for: Complexome profiling of the Chlamydomonas psb28 mutant reveals TEF5 as an early PSII assembly factor
Source: Plant Cell. 2025 Mar 17;37(6):koaf055. doi: 10.1093/plcell/koaf055 (PMC12164590; doi:10.1093/plcell/koaf055)
Supplement: koaf055_Supplementary_Data [file koaf055_supplementary_data.zip › psb28_interactive.zip!/plots/intensity/ACC1_Cre12.g519100.t1.2.html]

|  |  |
| --- | --- |
| **Identifier** | Cre12.g519100 |
| **Trivial name** | ACC1 |
| **Euclidean distance** | 2420.69 |
| **Average intensity sum (WT)** | 278.9 || **Average intensity sum (Mut)** | 225.5 |
| **Adjusted euclidean distance** | 8.68 |
| **MapMan annotation** | secondary metabolism.unspecified lipid metabolism.FA synthesis and FA elongation.acetyl CoA carboxylation.heteromeric complex.alpha carboxyltransferase |
| **p-value** (Welch test) of intensity sums | 0.56905 |
